# Clinical evaluation of inter-implant distance influence on the wear characteristics of low-profile stud attachments used in mandibular implant‑retained overdentures

**DOI:** 10.4317/jced.55433

**Published:** 2019-01-01

**Authors:** Nesreen El Mekawy, Mohamed-Yosry Elhawary

**Affiliations:** 1 BDS, MSc, Phd. Associate professor of Removable Prosthodontics, Faculty of Dentistry, Mansoura University, Mansoura, Egypt; 2 Clinical Demonstrator of Oral and Maxillofacial Surgery, Faculty of Dentistry, Mansoura University, Mansoura, Egypt

## Abstract

**Background:**

This study was aimed to evaluate the influence of inter-implant distance on the wear characteristics of low-profile stud attachments used in mandibular implant retained overdentures.

**Material and Methods:**

Forty Completely edentulous participants aged between 50 – 70 years were enrolled in this study. Each patient received 2-implants by 2-stage submerged surgical protocol. Participants categorized into 4-groups. Group I: 19 mm inter-implant distance with Locator retained overdentures; Group II: 19 mm inter-implant distance with OT Equator retained overdentures; Group III: 25 mm inter-implant distance with Locator retained overdentures; Group IV: 25 mm inter-implant distance with OT Equator retained overdentures. The female housings of each attachment were picked up to the mandibular overdenture. 12 month later the male inserts were replaced by new one. The used retentive male inserts were examined by Stereomicroscopic.

**Results:**

Stereomicroscopic examination revealed wear were detected on both inner surface and, the core of male inserts. Comparison between the unused and the used Locator and OT equator retentive male inserts at various inter-implant distance revealed highly significant wear changes between them at either 19 mm, or 25 mm inter-implant distance P1= .000, P2=.000 respectively.

**Conclusions:**

After one year of implant overdenture clinical use; both locator and OT equator retentive male inserts revealed significant surface deformities and wear. Wear were more notable on both locator and OT equator retentive male inserts with 25 mm interimplant distance than with 19 mm interimplant distance.

** Key words:**Inter-implant distance, attachments wear, locator retentive male inserts, OT equator retentive male inserts.

## Introduction

People with total edentulism has been considered by the World Health Organization (WHO) as physically handicapped people (1). In the past decades, complete dentures have been offered to edentulous people as the best treatment method for their disability; in the same time, edentulous people have many perpetual problems wearing complete denture. It is known that function impairment, denture looseness, unfavorable esthetic, and patient discomfort are difficulties associated with complete dentures (2). Indeed, the introduction of dental implants to be used in conjunction with the complete denture has proved many of troubles associated with that denture. As the dental implants represent successful and prospective treatment modality to the edentulous patients. Actually; implants would improve the denture support, retention and stability that in turn ameliorate patients’ satisfaction (3). Moreover, it has been revealed that; people with dental implants treatment provide almost double biting forces than that with complete denture (4,5).

An edentulous mandible could be restored with an overdenture constructed with varying numbers and positions of the dental implant (6). It was reported that; the utilization of 2 interforaminal dental implants with the lower denture provide predictable and, reliable treatment consequences (2,3,7,8). Additionally; according to the York (9) and, the McGill (10) Consensus Statements, the using of overdenture on 2 implants was proposed as a gold standard care for complete edentulous mandible. (11) The advantages of this implant rehabilitation kind are: a) provide good retentiveness ensuring stability to the prosthesis; (12) b) improve facial support particularly with severely resorbed ridge; c) less expensive as it utilize only 2 implants; d) preferable oral hygiene procedures due to easy removal of the prostheses; e) it provide good occlusal stability for the opposed prostheses (13).

Numerous attachment systems are utilized to connect the implants with the overdentures, these attachments categorized into splinted and unsplinted (14). Many factors play important role in the selection of the retentive mechanism and, planning the design of the implants retaining overdenture. Jaw morphology, the available vertical and horizontal prosthetic space, the alignments of the implants, the complexity of the case, the retention value needed, (15,16) mastication forces, cost of the treatment and, capability of load distribution are some of these factors. (17-19) Several randomized clinical trials (6-8,20), that discuss the implant overdenture, do not clearly specify the precise position of the implants, inserted into the interforaminal region (20). Nevertheless; the interimplant distance is a key factor in the designing of the overdenture as it may cause restriction of dental implant placement in the desired position and angulation, also it influence the overdenture retention. In addition, its effect on the attachment is poorly documented. Other studies (21,22) specify the minimum interimplant distance is 16 mm the always utilized distance is19 mm as the minimum while the maximum distance is 31 mm, a 29 mm value was used to better account for anatomic limitations such as the curvature of the mandibular arch and the position of the mental foramina.

Among the unsplinted systems used for connection, Locator attachment gained popularity among the low profile stud attachment systems, it has a dual retention approach, self-aligning attachment (23). This attachment is testify to good clinical performance, which improved patient quality of life (24). Nonetheless, excessive wear, increased maintenance requirements, decline in the retentive capacities, loss of retention have been intermittently described as diverse clinical behavior (25,26). Other recently utilized low profile stud attachment is the OT Equators system that combine the simplicity of Ball Attachments, with the variety of retention levels and easy replacement options of Locators. However OT-Equators offer many totally unique design benefits that the other systems lack: A significantly lower height and smaller diameter, hygiene-friendly construction and simple affordability to name a few (27).

Wear is known as “loss of material from a surface produced by various factors that are mechanical alone or through a combination of chemical and mechanical actions.” Attachment systems inevitably wear during function (28). Stereomicroscopic photographs is one of the observational method that confirm the presence of the surface material loss of the attachments (29). According to the author knowledge there is a lack of documentation about the influence of different interimplant distance on the low profile attachment system. Hens; this study was aimed to evaluate the influence of inter-implant distance on the wear characteristics of low-profile stud attachments used in mandibular implant retained overdentures.

## Material and Methods

This observational prospective study protocol and methodology were reviewed and approved by the Dental Research Ethical Committee of faculty of dentistry, Mansoura University. In addition; the study was performed in adherence to all ethical principles and has been in compliance to the STROBE (Strengthening the Reporting of Observational Studies in Epidemiology) standards/checklists according to von Elm *et al.* (30).

-Study Design and Setting

The study was conducted between February 2015 and December 2017. During this period; Patients had their implants, osseointegrated, loaded and, had their prostheses for a minimum of 1- year.

-Participants

Forty participants enrolled in this observational prospective study were from the prosthodontics department out-clinic. They were enrolled according the following criteria: Completely edentulous patients, aged between 50 – 70 years, have mandibular intra-foraminal distance more than 25 mm, 21,22 sufficient bone quantity (31) and quality (32) bilaterally in the mandibular canines area to install 2 implants of minimum 11.5 mm length, 3.5 mm diameter. Participants were excluded from this study if they unwillingness to sign the consent form, have osteoporosis, uncontrolled diabetes mellitus, smokers, alcoholism or have radiotherapy, chemotherapy, need for major bone augmentation procedures.

-Participants groups:

The enrolment participants were assigned into one of the four study groups according to the intra-foraminal distance present, no randomization was performed. Group I: included 10 participants with 19 mm intra-foraminal distance and, received Locator retained overdentures, group II: included 10 participants have 19 mm intra-foraminal distance and, received OT equator retained overdentures, Group III: included 10 participants with 25 mm intra-foraminal distance and, received Locator retained overdentures, Group IV: included 10 participants with 25 mm intra-foraminal distance and, received OT equator retained overdentures.

**Figure 1:**
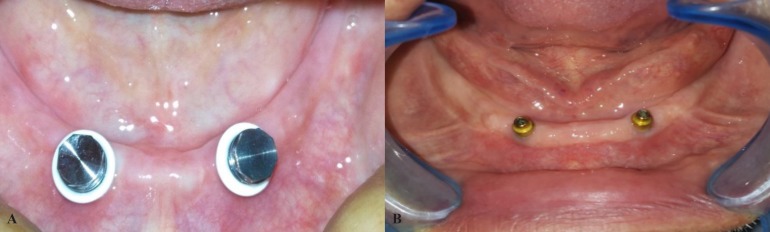
A. Locator metal Housing with white block out spacer in the patient mouth. B. OT equator attachment in the patient mouth.

-Surgical and prosthetic procedures:

For all participants new maxillary and mandibular complete dentures were constructed with balanced occlusal bilateral contact. The participants were pliable to use the denture for at least two months in order to improve the muscles adaptation to the denture. A stereolithography surgical template was constructed for each participant by using computed tomography (CT) (33) scans to be used in the implants installation. Two implants (Neobiotech Dental Implants, KOREA) were inserted bilaterally in the canine area according to the predetermined intra-foraminal distance of each patient using standardized 2-stage submerged surgical protocol. Mandibular denture was relined by tissue conditioner (Ufigel; Voco, Cuxhaven, Germany). Three months after implant installation by help of implant guide, circular tissue punch to remove a circular incisions at location of each fixture. Healing abutments were secured to each implants to allow the mucosa healing around the abutment. Two weeks later; either Locator (Locator© Zest anchor, USA) or OT equator (O T Equator, Rhein83, ITALY) attachments (Fig. 1 A,B) of appropriate gingival height were secured to the implants according to the corresponding group of the study using 30 Ncm torque. For all participants; the female housings of each attachment were picked up to the intaglio surface of the mandibular overdenture after relining intraorally by using auto-polymerized acrylic resin (Self cure acrylic resin, Acrostone Dental Factory, Egypt). Denture base acrylic resin around the attachments were relived sufficiently (about 0.5 mm) to avoid any interference that may increase the stresses around the implants. All prosthetic procedures were done by the same investigator. Occlusion were adjusted after relining. Instructions of performing good hygiene were provided to all participants. Each participant was commanded to leave the denture out at night. All participants were scheduled for follow-up visits.

**Figure 2:**
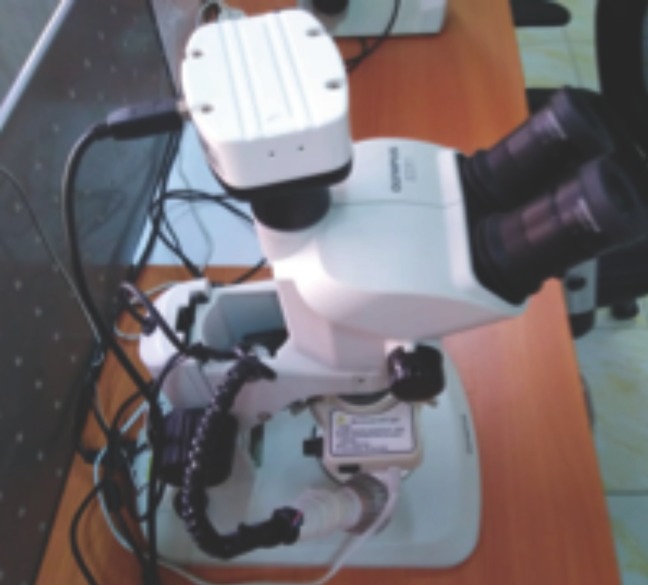
Stereomicroscopic.

According to the follow-up schedule; 12 month later the participants complaints from diminishing of the overdenture retention. By clinical examination, it was found that the retentive male insert of the various attachments were need to be replaced by new retentive male inserts to restore the retention of the experimental overdentures. The used retentive male inserts were examined by Stereomicroscopic (Olympus SZ61TR Trinocular Zoom, USA) (Fig. 2) to assess the wear characteristics of them.

-Microscopic measurements

The 80 specimens examined by Stereomicroscopic 40 Locator attachments and, 40 OT equator attachments retentive male insert were photographed using a 20 mega pixel digital camera with a ring flash to obtain a suitable light conditions. The units were placed so the central nylon core was accurately perpendicular to the lens axis and fully in focus; by using a mounting device located at a fixed distance from the optical plane. Obtained images were transferred to a widows 7® based computer for further analysis.

-Digital Image Analysis 

Analysis was performed using VideoTest® morphology® (Russia) by the following steps.

• Outline was manually extracted using genius® digital pen tablet to obtain the shape outline;

• Color levels of the images were adjusted to enhance the variation between normal and damaged surface, then converted to grayscale images.

• Images were threshold at the level of the damaged areas depending on variation in gray scale and represented as region of interest (ROI) for which areas were measured;

• Area percent of damaged areas was calculated using steps 1 and 4. Results were exported to Excel® sheet for further statistical analysis

-Statistical analysis 

Percent of damaged areas was confirmed by Shapiro-Wilk’s test (*p* > 0.05). Independent Samples Test was used to compare between various groups of the study. While, One-way were used to compare between the unused and the used retentive male insert of both attachments types at different interimplant distance. The SPSS statistics 20.0 statistical package (IBM, Armonk, NY) was used for all statistical analyses.

## Results

**Figure 3:**
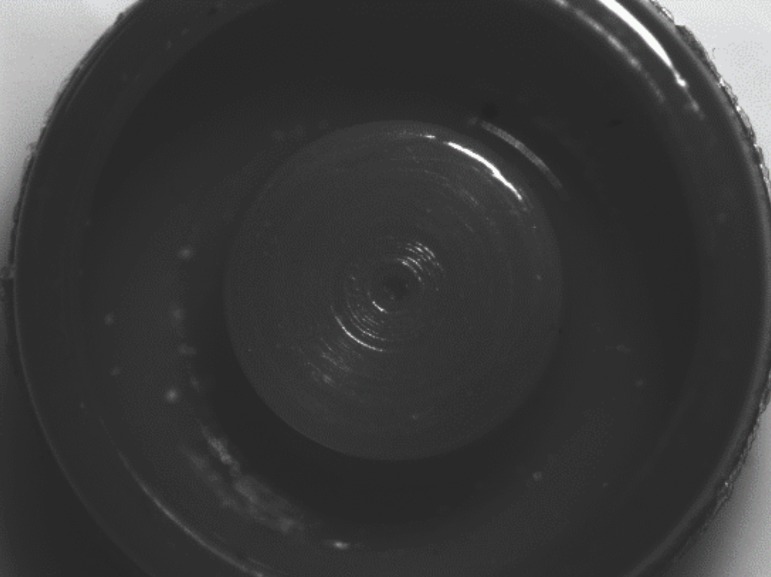
Photomicrograph of the unused Locator attachment retentive male insert.

**Figure 4:**
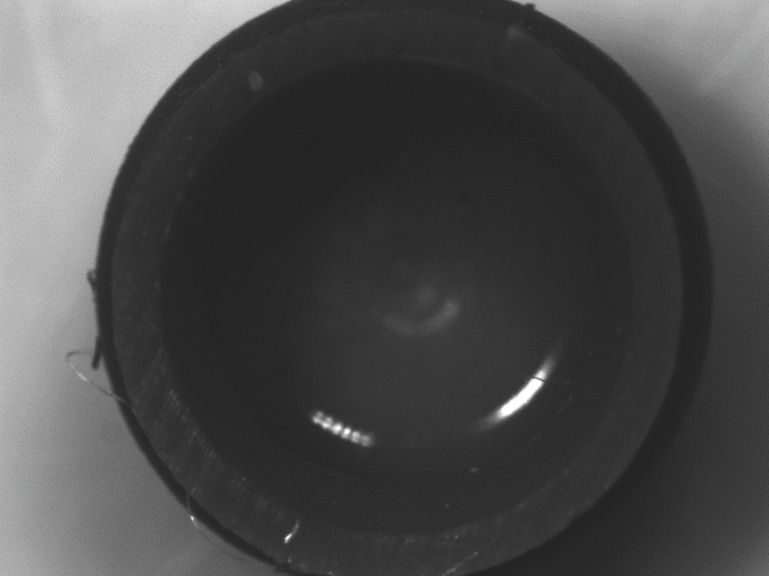
Photomicrograph of the unused OT Equator attachment retentive male insert.

**Figure 5:**
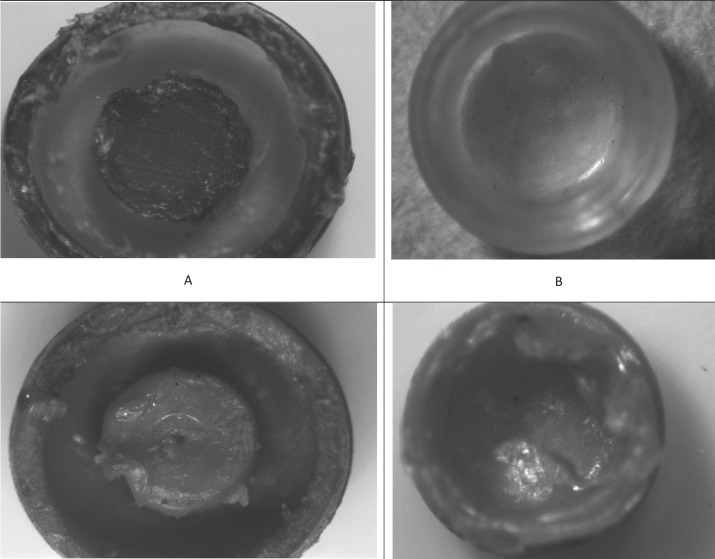
Photomicrograph of the used retentive male insert of locator and OT equator attachment systems; A. Locator retentive male insert at 19 mm interimplant distance have inner surface deformity scratches of the central core; B. OT equator retentive male insert at 19 mm interimplant distance have wear of inner surface deformity scratches of the central core; C. Locator retentive male insert at 25 mm interimplant distance have sever inner surface deformity deterioration of the central core; D. OT equator retentive male insert at 25 mm interimplant distance have sever wear and loss of surface particles of inner surface, deformity and scratches of the central core.

Stereomicroscopic examination revealed surface characteristics of the used retentive male insert and the unused ones. The unused retentive male insert of both attachment types’ photos revealed a smooth, finely grained inner surface as represent in (Figs. 3,4). The used locator and OT equator retentive male inserts photos with different interimplant distances were presented in (Fig. 5); the wear characteristics were detected on both inner surface and the core of retentive male inserts. Wear effects were more notable on both locator and OT equator retentive male inserts retentive male inserts with 25 mm interimplant distance. Scratches, surface alterations, micro-voids, and particle loss were obviously appears on the inner surface of the used inserts.

Comparison between the unused and the used Locator and OT equator retentive male inserts at various inter-implant distance were represent in  Table 1 and,  Table 2 respectively. In case of Locator male inserts in  Table 1, there were highly significant wear changes between the unused and the used male inserts at either 19 mm, or 25 mm interimplant distance P1= .000, P2=.000 respectively. While; comparison between the used Locator male inserts at 19 mm interimplant distance and 25 mm interimplant distance have high significant wear as P3=.000.

**Table 1:**
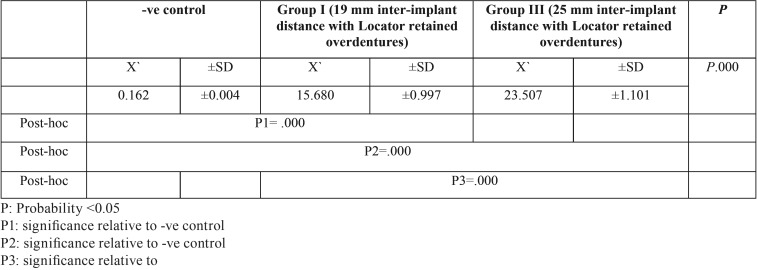
Comparison between the unused and the used Locator retentive male inserts at various inter-implant distance.

**Table 2:**
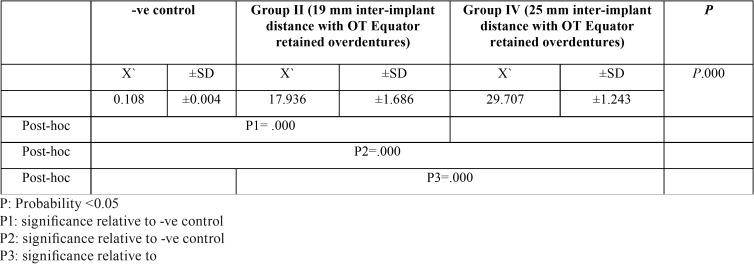
Comparison between the unused and the used OT Equator retentive male inserts at various inter-implant distance.

In case of OT equator male inserts in  Table 2, there were highly significant wear changes between the unused and the used male inserts at either 19 mm, or 25 mm interimplant distance P1= .000, P2=.000 respectively. While; comparison between the used OT equator male inserts at 19 mm interimplant distance and 25 mm interimplant distance have high significant wear as P3=.000.

 Table 3 revealed comparison between the used Locator and OT equator retentive male inserts at the 19 mm inter-implant distance, it was found significant wear changes between retentive male inserts of the two attachments system as P.002 and, P.003 respectively. While  Table 4 revealed comparison between the used Locator and OT Equator retentive male inserts at 25 mm inter-implant distance, , it was found significant wear changes between retentive male inserts of the two attachments system as P. 000 and, P.000 respectively.

**Table 3:**
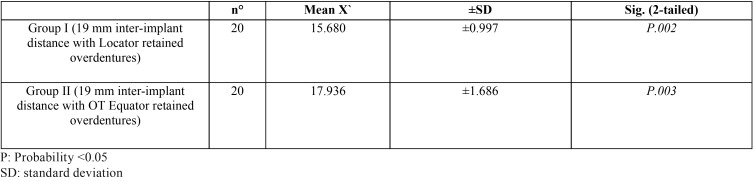
Comparison between the used Locator and OT equator retentive male inserts at the 19 mm inter-implant distance.

**Table 4:**
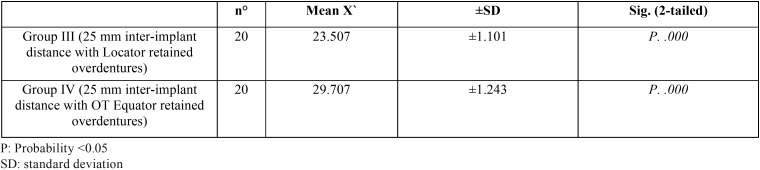
Comparison between the used Locator and OT Equator retentive male inserts at 25 mm inter-implant distance.

## Discussion

Many functional, anatomic, and psychosocial benefits were provided to edentulous patients by implant overdentures (34). Two intercanine implants with attachments used to retain the denture improves the biting force and increases stability of the mandibular overdenture that improve the quality of life of edentulous patients (35). A review of Alsabeeha (28) concluded that studying mandibular implant overdenture effective factors must be investigated separately to limit the impact of confounding variables on the end result. Present study accessed the effect of inter-implant distance on the surface changes and wear of a mandibular implant overdenture attachments.

The existing evidence base describing wear characteristics across various interimplant differences is limited, with the majority of the studies are in vitro study concentrating on the evaluation of retentive characteristics of locators on fixed or arbitrary interimplant distances only (19,36). Only a single clinical research has evaluate the effect of interimplant distance on mandibular implant overdenture; however, this research investigate The influence of inter-implant distance in mandibular overdentures supported by two implants on patient satisfaction and quality of life (37).

Overdenture attachments have to fit to big number of requirements as Proper retention, support, stability, ease of maintenance, and longtime service. So, there is no a straightforward superiority of an overdenture attachment type. Regardless the popularity of resilient overdenture attachments, there are lack of the studies which have investigate interimplant differences and the wear effects (19).

A collective observation in this study is the increasing of the surface deformity and wear of the nylon retentive male inserts of both locator and OT equator after one year of attachment function in the patient mouth. These finding of the study were overturn in patients complaining from diminishing of the denture retention. There is research and clinical evidences that overdenture retention properties of the various attachments tend to change with time (36) Function of resilient overdenture attachments is adversely affected by the wear. Thus, patient satisfaction and, maintenance aspects is paramount for the clinician.

The clinical wear of overdenture attachments is associated with several factors that include masticatory forces (38), the composition and temperature of saliva and, parafunctions. Moreover, denture cleansers used to maintain denture hygiene (39), as well as food residues (38), all these factors difficult to simulate *in vitro*. The tear and wear of retentive male inserts of an overdenture attachment system occurs typically in the first 12 months of use, increasing the need for periodic maintenance, 40 this is in coincidence with the current study.

The present study revealed that there was significant increase in the surface wear of the retentive male insert of both attachment systems between various evaluations at the begin of the study and after one year from overdenture insertion. This is contributed to the changes of the physical properties of the retentive male inserts as a result of the fatigue experienced during the clinical using of the attachments. Whether the patient would notice the loss of retention in the first few months of wear would be of interest to establish (41). This in agreement with Kleis *et al.* (42), who reported that the Locator attachment showed 75.5% losses of retentions because of the wear of the male parts, which made a change of these parts necessary. Also; Tomás *et al.* (43) explained in an *in vitro* study that both locator and OT equator behavior show similar patterns without statistically significant differences until the 7,500 th cycle.

As the retentive male inserts of both attachment systems were manufactured by nylon or plastic components due to their ease and cost of manufacturing, the higher wear may be due to the various geometries of the plastic matrices of the two attachment systems that may resulted in higher friction forces or higher wear resistance of the retentive male components. In addition, the unequal dimensions of the retentive male components of both attachment systems that may provoked this result (3).

Based on the results of several *in vitro* studies (20,21,38,44) installing two intraforaminal implants with more interimplant distance had advantageous in improving the resistance of functional forces; however, it is unknown what effect this has on load distribution to the implants and mucosa, or the wear changes of the attachment systems. The result of this clinical study revealed that increasing of the interimplant distance increase the surface changes and wear of the retentive male inserts of the two attachments system used in the study.

Noteworthy results of the Stereomicroscopic observations showed that wear effects were more notable on both locator and OT equator retentive male inserts with 25 mm interimplant distance than with 19 mm interimplant distance. This results in coincidence with Hong *et al.* (44), who showed, in a finite element study, that the placement of the implants more anteriorly, corresponded to 19 mm interimplant distance, reduce the peri-implant bone stresses so that the surface changes of the male inserts is less notable in this situation. In addition this provides a mechanical advantage for overdenture support (21,37). This in contrary to the result of Marin et al who reported that implant placement in the area of the lateral incisor result in occlusal complications leading to tipping of the prosthesis.

The findings of the current study, when comparing the wear of locator and OT equator retentive male inserts at the certain interimplant distance either 19 mm, or 25 mm, revealed that the wear of the OT equator male inserts were more remarkable than the wear of the locator attachment male inserts. This may be attributed to firstly; the difference in inner diameter of the retentive male inserts of two attachment systems, secondly; the presence of the central core in the locator retentive male inserts and the absence of them from the OT equator retentive male inserts (29).

The limitations of this clinical study, limited number of patient enrolled in this study that carried out on only two attachment systems; that was not enough to reveal all about wear phenomena in relation to interimplant distance; so that further studies are necessary for better evaluations to attachments introduced in the market. Further studies are suggested considering both factors: inter-implant distance and load distribution to implants. Furthermore, randomized clinical trials are needed to assess these factors in patients at longer follow-up period.

## Conclusions

The following conclusion were obtained within the limit of this study.

1. After one year of clinical use of implant overdenture; both the locator and OT equator retentive male inserts revealed significant surface deformities and wear.

2. Wear were more notable on both locator and OT equator retentive male inserts with 25 mm interimplant distance than with 19 mm interimplant distance.

3. Wear of the OT equator male inserts were more remarkable than the wear of the locator attachment male inserts at the certain interimplant distance either 19 mm, or 25 mm.
